# Histogenesis of retinal dysplasia in trisomy 13

**DOI:** 10.1186/1746-1596-2-48

**Published:** 2007-12-18

**Authors:** Ada Chan, Satyan Lakshminrusimha, Reid Heffner, Federico Gonzalez-Fernandez

**Affiliations:** 1 Department of Pathology, State University of New York, Buffalo, New York, USA; 2 Department of Pediatrics, State University of New York, Buffalo, New York, USA; 3 Ross Eye Institute and departments of Ophthalmology, Pathology, Biochemistry, State University of New York, Medical Research Service, Veterans Affairs Medical Center, Buffalo, New York, USA

## Abstract

**Background:**

Although often associated with holoprosencephaly, little detail of the histopathology of cyclopia is available. Here, we describe the ocular findings in a case of trisomy 13 to better understand the histogenesis of the rosettes, or tubules, characteristic of the retinal dysplasia associated with this condition.

**Methods:**

A full pediatric autopsy was performed of a near term infant who died shortly after birth from multiple congenital anomalies including fused facial-midline structures. A detailed histopathological study of the ocular structures was performed. The expression of interphotoreceptor retinoid-binding protein (IRBP), cellular retinal-binding protein (CRALBP), rod opsin, and Sonic Hedgehog (Shh) were studied by immunohistochemistry.

**Results:**

Holoprosencephaly, and a spectrum of anatomical findings characteristic of Patau's syndrome, were found. Cytogenetic studies demonstrated trisomy 13 [47, XY, +13]. The eyes were fused but contained two developed separate lenses. In contrast, the cornea, and angle structures were hypoplastic, and the anterior chamber had failed to form. The retina showed areas of normally laminated neural retina, whereas in other areas it was replaced by numerous neuronal rosettes. Histological and immunohistochemical studies revealed that the rosettes were composed of differentiated retinal neurons and Müller cell glia. In normally laminated retina, Shh expression was restricted to retinal-ganglion cells, and to a population of neurons in the inner zone of the outer nuclear layer. In contrast, Shh could not be detected in the dysplastic rosettes.

**Conclusion:**

The histopathology of cyclopia appears to be more complex than what may have been previously appreciated. In fact, the terms "cyclopia" and "synophthalmia" are misnomers as the underlying mechanism is a failure of the eyes to form separately during development. The rosettes found in the dysplastic retina are fundamentally different than those of retinoblastoma, being composed of a variety of differentiated cell types. The dysplastic rosettes are essentially laminated retina failing to establish a polarized orientation, resulting in the formation of tubules. Finally, our findings suggest that defective ganglion cell Shh expression may contribute to the ocular pathology of cyclopia.

## Background

The retina is a striking example of architectural polarity. At its vitreal surface, it is bordered by the retinal-ganglion cells, and sclerad by the retinal pigmented epithelium (RPE). Adjacent to the RPE, photoreceptor nuclei comprise the outer nuclear layer; a variety of interneurons and Müller glia make up the inner nuclear layer, which is adjacent to the ganglion-cell layer. The ganglion and RPE cell layers, which are the first layers to differentiate, establish the polarity of the remaining layers during development. In some pathological states, this ordered arrangement is disrupted resulting in "retinal dysplasia", which is characterized by tubular structures known as "rosettes". Such rosettes are reminiscent of the Flexner-Wintersteiner rosettes and fleurettes of retinoblastoma. However, unlike their malignant counterpart, dysplastic rosettes do not represent a neoplastic, or even pre-neoplastic process. Little is known about the histogenesis of dyplastic rosettes. Cyclopia provides an ideal setting to study retinal dysplasia as rosettes are common in this condition.

Cyclopia may be associated with holoprosencephaly, the most common developmental defect of the forebrain with an incidence of 1:250 during embryogenesis. Due to intrauterine lethality, live born prevalence of holoprosencephaly is 1:16,000. It can range from major brain and facial anomalies to clinically normal individuals having only a single fused central incisor [reviewed in [1-5]]. Holoprosencephaly is a malformation sequence in which impaired midline cleavage of the embryonic forebrain is a fundamental feature [6-11]. The prosencephalon fails to cleave sagitally into cerebral hemispheres, and transversely into telencephalon and diencephalons. Given the complexity of the cellular interactions occurring during forebrain development, it is not surprising that a variety of genes and teratogens have been implicated in the pathogenesis of holoprosencephaly [2,3,6,7,11-22].

The study of ocular development is providing insight into the pathogenesis of holosproencephaly. It is now appreciated that during development, a single eye field is bisected by signals from the underlying prechordal mesoderm. The early eye field therefore represents a larger area of neural ectoderm that is competent to respond to the inducer signal to form an eye. If the competence of the central eye field is not inhibited when induction begins, the entire field will respond with the formation of a single eye. This inhibition appears to be mediated by Sonic Hedgehog (Shh). Mutational inactivation of Shh, or its receptor Ptch, can result in holoproscencephaly with cyclopia [7,9,12,13,15,18,22-26].

Although cyclopia is well documented clinically, detailed understanding of the consequences of cyclopia to ocular development is limited [27]. Here, we hope to extend our understanding of human cyclopia in the setting of holoprosencephaly with particular attention to the histogenesis of retinal dysplasia, a poorly understood yet common ophthalmic pathological finding [28].

## Results

### Clinical history and general autopsy findings

The infant was born at 37 2/7 weeks gestation to a 19 year old African-American mother. She had one previous pregnancy, a boy 4 years old with no congenital anomalies. She denied taking alcohol, tobacco, recreational drugs, or any medications except for multivitamins. She had three prenatal visits including an unremarkable ultrasound at 15 weeks gestation. A cervical culture was positive for group B streptococcus. Cesarean section delivery was performed due to clinical evidence of fetal distress. The baby had multiple congenital anomalies including an occipital encephalocele, proboscis, cyclopia, bilateral post-axial polydactyly and a two-vessel cord. Apgar scores were 1, 2, and 4 at 1, 5, and 10 minutes respectively. The baby required intubation for apnea and was transfered to the neonatal intensive care nursery. Given the severe nature of the congenital defects, and the impossibility for survival, the family decided to withdraw life support.

**Figure 1:**
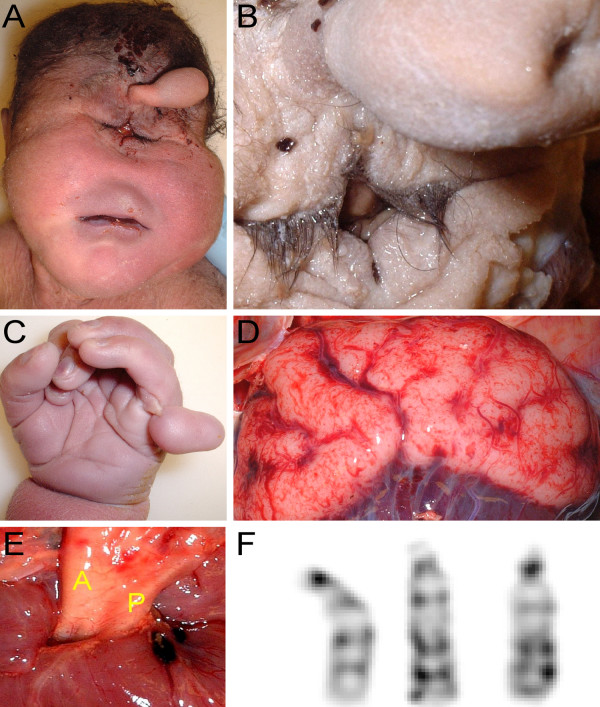
**A 37 2/7 week gestational age male infant with Patau syndrome demonstrating alobar holoprosencephaly with cyclopia**. **A) **Facial features included sloping forehead with a proboscis superior to a single central palpebral fissure. **B) **Close-up of the fused eyelids and proboscis showing a single nostril. **C) **Polydactyly showing six digits. **D) **Posterior view of the brain showing indistinct gyri, fusion of the hemispheres, and occipital encephalocele.**E) **Transposition of the aorta (A), and hypoplastic pulmonary trunk (P). **F) **Trisomy 13 [47, XY, +13] (karyotype by Giemsa-banding).

Complete autopsy was performed after an informed consent. The autopsy showed a well nourished baby boy of normal height and weight, having a short neck, sloping forehead, and low set ears (Fig. 1A). Midline facial abnormalities included a proboscis with single nostril arising above a pair of fused eyelids (Fig. 1B). The lips and palate were intact. Rudimentary sixth digits were present on the right and left hands, and the feet were broad and flat (Fig. 1C). The spine was intact with a sacrococcygeal skin tag.

Microcephaly was present (23.5 cm in circumference; normal, 32.7 ± 5.1 cm) with an occipital encephalocele extending bilaterally to the parietal lobes (Fig. 1D). The brain appeared asymmetric showing two fused cerebral hemispheres. The gyrational pattern was not developed. Coronal sections of the cerebrum showed no division into two separate hemispheres. The cerebral gyri were immature without evidence of microgyria, or other cortical malformations. One common ventricle without division into paired frontal and occipital horns was noted. The basal ganglia could not be identified. One thalamus with a small "third" ventricle at the center was present. The cerebellum and brainstem were unremarkable except for fusion of the inferior cerebellar hemispheres in the region of the nodulus and uvula. The olfactory nerves were not identified and olfactory bulbs and tracts were not present. Microscopic examination showed a malformed cortex at the site of fusion of the cerebellar hemispheres, with a haphazard arrangement of all three layers. The outer layer of the thalamus was disorganized and malformed, resembling the tissue of the optic nerve.

The cardiac apex pointed to the left. The aorta arose from the right ventricle and lay anterior and to the right of a stenotic and hypoplastic pulmonary artery (Fig. 1E). Large atrial and ventricular septal defects were found with a communis defect involving the endocardial cushions. The right lung had three lobes that showed extensive atelectasis; the left lung had two lobes and was partially aerated. There were no pathologic findings in the gastrointestinal and genitourinary systems except for malrotation of the large bowel (cecum and ascending colon were on the left side of the abdominal cavity).

Taken together, the anatomic findings are consistent with Patau's syndrome with holosprosencephaly. This was confirmed by the karyotype analysis, which showed trisomy 13 (47, XY, +13) (Fig. 1F).

### Ocular pathology

**Figure 2:**
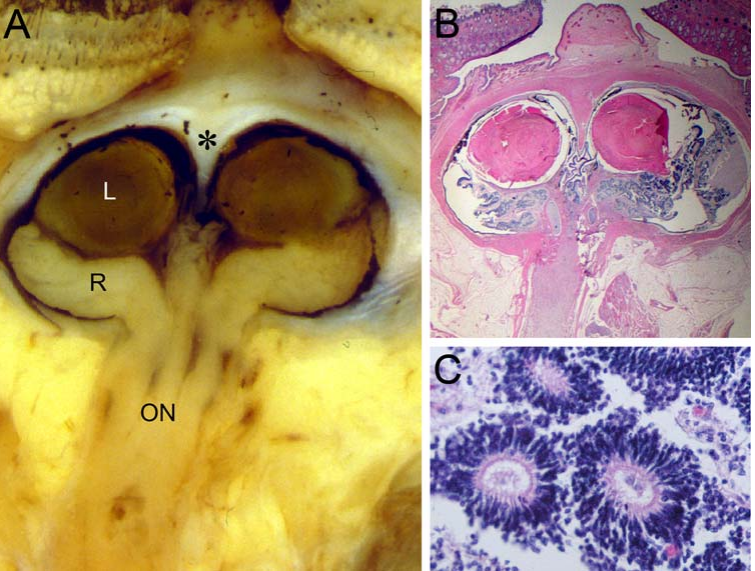
**Transverse section of the fused eyes**. **A) **Gross view showing two separate lenses (L), retina (R), and partially fused optic nerve (ON). An incomplete septum extends posteriorly from the anterior sclera (asterisk). **B) **Histological section demonstrating a single common posterior chamber containing laminated and disorganized neural retina. **C) **Higher magnification of the disorganized retina showing dysplastic rosettes (hematoxylin and eosin B, C).

The globes and eyelids were fused at the midline. The continuity of the two superior, and the two inferior lids created a rhomboid-shaped midline palpebral fissure inferior to the proboscis (Fig. 1B). Beneath the lids, anterior eye structures including a transparent cornea and the pupil were not found. A horizontal transverse section through the eyes is shown in Fig. 2A. The eyelids contained well-developed structures including meibomian glands and hair follicles (Fig. 2B). The inner palpebral surfaces and the sclera-like tissue over the eyes were covered by conjunctival epithelium containing numerous goblet cells. The histological sections oriented through the pupil showed a striking absence of corneal differentiation. Corneal epithelium, stroma, or Descemet's membrane could not be identified. Instead, underlying the anterior connective tissue was a mesh-like loose band of tissue covering the front of the anterior chamber. Although RPE was identified, the angle, trabecular meshwork, and iris had failed to form.

The gross and histological sections showed a single globe incompletely divided into separate eyes by a partial fibrous septum. The septum extended posteriorly from the anterior sclera separating the two lenses (asterisk, Fig. 2A,B). However, it failed to extend sufficiently in the posterior direction to finish dividing the rest of the eye into separate chambers. The partition was lined by the RPE layer. In some areas the retina showed a near normal laminar structure consisting of well defined inner and outer nuclear layers. However, most regions were disorganized consisting of scattered rosette-like structures (Fig. 2C). A focal region of cartilage and CNS tissue were also found in the dysplastic retina. The retina collected at a single point at the posterior pole forming a fused single hypoplastic optic nerve. The rosettes extended into the optic nerve head, and to some degree down the optic nerve itself. Although there appeared to be one optic nerve, some of the sections suggest the presence of two incompletely separated nerves.

**Figure 3:**
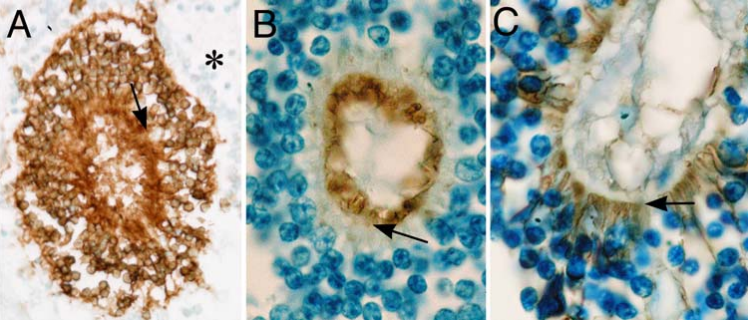
**Immunoperoxidase localization of photoreceptor and Müller cell markers in the rosettes**: **A) **The majority of cells comprising the central zone of the rosettes were positive for rod opsin. Cells in the peripheral zone were negative (asterisk). **B) **interphotoreceptor retinoid-binding protein (IRBP), which is normally secreted by photoreceptors, was restricted to the peripheral lumen of the rosette inside of the external-limiting membrane (arrow). **C) **Occasional cells showed Müller cell differentiation expressing cellular retinaldehyde-binding protein (CRALBP) positive radial processes that abruptly end at the external limiting membrane. Sections treated with non-immune serum showed no immunospecific reactivity (data not illustrated). Arrows in each panel indicate the position of the external-limiting membrane.

**Figure 4:**
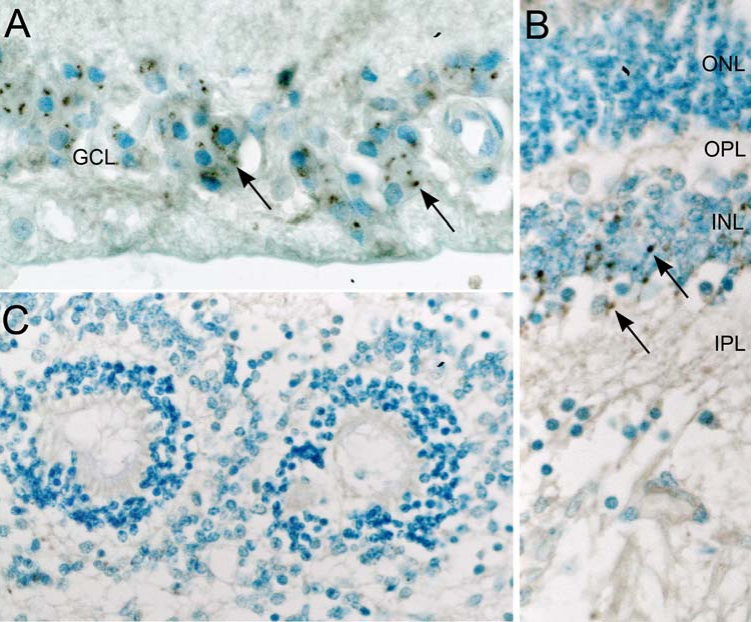
**Immunoperoxidase localization of sonic hedgehog (Shh) in the normal human infant retina, and dysplastic retina**. **A) **Ganglion cell layer from a region of normal retina of an infant with retinoblastoma. Shh is localized in the ganglion cell soma as punctate cytoplasmic staining (arrows). Shh was not detected in other regions of the retina, or in sections similarly treated with non-immune serum (data not illustrated). **B) **Region of normally laminated retina from the present case showed cytoplasmic shh in neurons residing in the inner nuclear layer (arrows). **C) **The region comprising dysplastic rosettes did not stain for Shh. Outer nuclear layer (ONL), outer plexiform layer (OPL), inner nuclear   layer (INL), inner plexiform layer (IPL), and ganglion cell layer (GCL).

**Figure 5:**
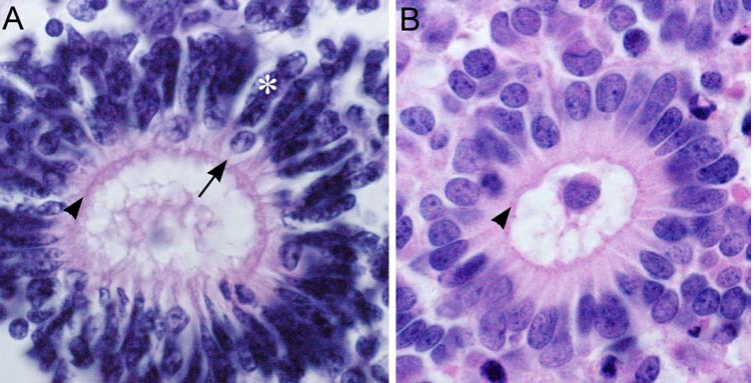
**Comparison of dysplastic rosettes to the neoplastic rosette of retinoblastoma**. **A) **Rosette from area of dysplastic retina in the present case.**B) **Flexner-Wintersteiner rosette from a case of retinoblastoma. Short arrows, external limiting membrane; long arrow in A, possible cone nuclei; asterisk, possible rod nucleus.

Immunohistochemical studies were carried out to further characterize the rosettes (Figs. 3, 4). Most of the rosettes appeared to consist of two concentric zones recapitulating the outer and inner nuclear layers of the normal retina. However, the rosettes were inside-out compared to the normal retina in that the outer nuclear layer was more central to the inner nuclear layer. The lumens of the rosettes were often lined by an outer-limiting membrane (short arrows in Figs. 3, 5). The majority of cells comprising the central-nuclear zone of the rosettes were positive for rod opsin (Fig. 3A). Cells in the peripheral zone were negative (asterisk in Fig. 3A). Interphotoreceptor retinoid-binding protein (IRBP), a protein normally secreted by rod and cone photoreceptors [29], was found in the luminal wall of the rosettes inside or central to the outer-limiting membrane (arrow, Fig. 3B). Immunohistochemical localization of cellular retinaldehyde-binding protein (CRALBP), a retinoid binding protein normally expressed by Müller glia, is shown in Fig. 3C. Cells with Müller cell differentiation expressed CRALBP, and showed positive radial processes abruptly ending at the outer-limiting membrane of the rosette lumen (Fig. 3C).

In view of the central role of Hedgehog signaling in the pathogenesis of holoprosencephaly and cyclopia in particular, we wanted to define the distribution of Sonic Hedgehog (Shh) in the retina of the present case. As a positive control, we used normal human retinas from the eyes of infants enucleated for retinoblastoma. In these retinas, Shh could be identified in the retinal-ganglion cells, and in some of the neurons residing in the inner zone of the inner nuclear layer. In Fig. 4A, which corresponds to a region of normal retina from a child treated surgically for retinoblastoma, ganglion-cell Shh expression appears as punctate cytoplasmic staining. A similar staining pattern could be identified in regions of normally laminated retina in the present case (Fig. 4B). In contrast, wherever retinal rosettes were present, Shh staining was absent (Fig. 4C). The normally laminated retina provides an internal positive control indicating that the absence of Shh staining in the rosettes was not a technical artifact.

Figure 5 compares the structure of a rosette from the dysplastic retina with a typical Flexner-Wintersteiner rosette characteristic of retinoblastoma. Both rosettes contain an outer limiting membrane (short arrow) lining a central lumen. In contrast to the neoplastic rosette of Flexner-Wintersteiner rosette, the rosettes of the dysplastic retina contain multiple cell types as was shown above by immunoperoxidase staining, and even here in this hematoxylin & eosin stained section where the long arrow in panel A identifies a probable cone cell nucleus among numerous rod cell nuclei (asterisk). Furthermore, the rosettes in retinoblastoma are typically positive for cone opsin, and negative for rod opsin [30]. The dysplastic rosettes show the opposite pattern expressing mainly rod opsin. Finally, Müller cell differentiation is not observed in the rosettes of retinoblastoma from surgical specimens [30].

## Discussion

A common thread in the constellation of features found in this case is the failure of structures normally bilaterally positioned in the term neonate to separate from the midline during development. This is seen, for example, here in the failure of the brain to establish lateral hemispheres and in the globes to become separate eyes. The retained fusion of bilateral structures may be regarded as a defect in the establishment of polarity. In view of the increasing appreciation of embryonic induction and Hedgehog signaling in these processes, we wanted to explore whether such mechanisms could be extended to the tissue level allowing for an explanation of the ocular changes seen in human cyclopia.

The autopsy findings are consistent with the diagnosis of Patau's syndrome. This conclusion is confirmed by the cytogenetic demonstration of trisomy 13 in the patient. The etiology of trisomy 13 is unknown. The clinical presentation is typical except for the young age of the mother (19 years). Trisomies tend to occur with greater frequency with advanced age [31]. The age effect is not as great with trisomy 13 compared to 18 and 21 [32]. Trisomy 13 is more common in females [33]. It should be noted that in contrast to trisomy 21, the risk for recurrence of trisomies 18 and 13 is considered to be very low [34]. The phenotype in this case represents a more severe end of the spectrum with the manifestation of holosproencephaly and cyclopia.

In theory, cyclopia could result from the fusion of two originally separate eyes, or from the failure of a single primordium to separate during development. Microdissection studies have shown that removal of the prechordal mesoderm leads to the formation of a single retina in chick embryos and *Xenopus *explants [35,36]. For example, removal of the prechordal plate results in fusion of the forebrain as well as the retina [36]. Furthermore, the prechordal plate expresses Shh and was able to rescue the cyclopic phenotype in transplantation studies. Interestingly, transplantation of the prechordal plate to the vicinity of the optic cup suppresses the expression of *pax6 *in the retina. We can conclude that there is a single retinal morphogenetic field that resolves into two retina primordial. This is accomplished by a prechordal-plate signal that suppresses retinal formation in the medial region of the field [36]. This model is consistent with that suggested more than 75 years ago by Adelmann (1929) [37].

In the present case, a disruption of the normal sequence of development appears to have occurred after lens induction, but before the formation of the cornea and angle structures, suggesting an interruption of neural crest migration. Normally, the corneal stroma, endothelium, anterior iris, and trabecular meshwork are formed by neural crest cells entering the optic-cup lip. Although the reason for the defective neural crest migration is unknown, it is interesting that inhibition of Shh *in vivo *results in neural crest cell death [10]. The absence of the corneal endothelium, which promotes formation of the anterior chamber, could explain the lack of separation of the lens from the cornea [38].

Insight into the role that abnormal Hedgehog signaling may have in the histogenesis of retinal dysplasia has come from studies of *Ptch*. *Ptch *mutations cause Gorlin syndrome, an autosomal dominant disorder characterized by dental, skeletal and radiographic abnormalities including falx calcification, bifid/fused ribs and altered vertebral segmentation, and a predisposition to tumor development including early-onset basal cell carcinomas [39-44]. Ocular abnormalities are often present as were in the first patient described by Dr Gorlin [40]. These findings include microphthalmia, coloboma, cataract, inappropriate retinal myelination, and retinoschisis [45-50]. It is therefore intriguing that the Hedgehog pathway has not only a role in establishing separate eyes, but also in the development of the retina itself. This is further supported by studies in zebrafish indicating that signals from the prechordal mesoderm can effect retinal neurogenesis [51-53].

Our finding that Shh is found in the cytoplasm of the retinal-ganglion cells in humans is consistent with recent studies showing that its mRNA is expressed in the ganglion cells of other vertebrates [52,54-56]. Our finding of Shh positive cells in the inner nuclear layer is consistent with its known expression in zebra fish amacrine cells [55]. The significance of these observations is that Shh secretion by the ganglion cells appears to be critical to orchestrating the normal organization of the developing neural retina [52,54,55,57-61]. This notion is supported by the fact that *ptch *and *gli *are expressed in retinal neuroblasts, and astrocyte precursor cells in the optic nerve [62,63]. Retinal-ganglion cell derived Shh expression is required for Hedgehog target gene induction in the retina and optic nerve [56-58].

Notwithstanding the potential importance of the ganglion cells in these processes, it should be pointed out that the system is complex with other local sources and targets of *Hh *signaling [64,65]. In mouse, *Ihh *is expressed by RPE, and recombinant Hh protein promotes photoreceptor differentiation in vitro [57]. In zebrafish, *Shh *and *Twhh *are both expressed by RPE, and *Hh *signaling knockdown results in reduced photoreceptor differentiation, and retinal cell death [65,66]. Finally, the RPE itself may need *Hh *signaling in order to properly differentiate [67].

To address the question of the role of the Hedgehog pathway and the *ptch *receptor in particular on the development of the mammalian retina, Black et al (2004) [68] studied ocular development in mice heterozygotic for disruption of the *ptch *gene [69-72]. The retinas of PtchlacZ +/- mice exhibit abnormal cell cycle regulation, culminating in photoreceptor dysplasia and Müller cell gliosis. Interestingly, the PtchlacZ +/- mice also show vitreoretinal abnormalities resembling those found in patients with Gorlin syndrome and dysplastic rosette formation.

The histopathology of cyclopia appears to be more complex than what may have been previously appreciated. In fact, the terms "cyclopia" and "synophthalmia" do not reflect the histopathology or pathophysiology. True cyclopia, i.e., a single eye, is vanishingly rare, with most cases showing two fused eyes (synophthalmia). However, synophthalmia does not accurately reflect the mechanism resulting in the fusion of the eyes. That is, the fusion is not the result of two eyes coming together, but rather the failure of the eyes to form separately during development. According to the animal studies reviewed above, the defect is a failure of the Shh pathway to inhibit competence in the central eye field. The result is an incomplete separation of the eyes during development. At the tissue level, the complexity extends to a failure of the angle structures to form, and the retina to achieve its orientation toward the RPE.

Dysplastic rosettes are laminated retina failing to establish a polarized orientation toward the RPE resulting is the formation of tubules. The finding, which is not considered to be neoplastic or preneoplastic, is often associated with trisomy 13, but may be seen in other clinical [28] and experimental settings. The rosettes should be distinguished from the various rosettes seen in retinoblastoma which do not contain Müller cells [30,73,74]. The rosettes found in the dysplastic retina are fundamentally different than those of retinoblastoma, in that unlike their neoplastic counterpart they are composed of a variety of differentiated cell types.

The mechanism responsible for the histogenesis of retinal dysplasia is far from clear. The application of the term "retinal dysplasia" to describe a "maldevelopment of the retina" and early descriptions of its rosettes have been reviewed by Lahav et al. (1973) [28]. It has been proposed that the rosettes may represent an abortive attempt at regeneration [75], or an abnormality in programmed cell death [73]. Although the mechanism for the formation of the rosettes remains unknown, it is interesting that retinal dysplasia including rosette-like formations are found in *Patch *+/- mice and in conditional ablation of *Shh *[61,68]. In the present case, offering a mechanism for the histogenesis of the retinal dysplasia should be done with caution. That is, we cannot with certainty establish a cause-effect relationship between the absence of Shh expression and the formation of the rosettes. For example, it is possible that the lack of Shh staining could simply be a secondary effect due, for example, to the absence of the cells producing the protein. Thus, although alternative models should be taken into account, our findings, taken together with the available literature, suggest the possibility that the dysplastic rosettes in human cyclopia result from a direct or indirect failure of Hedgehog signaling.

## Abbreviations

IRBP: Interphotoreceptor retinoid-binding protein;

CRALBP: Cellular retinaldehyde binding protein;

RPE: Retinal pigmented epithelium;

Shh: Sonic hedgehog.

## Competing interests

The author(s) declare that they have no competing interests.

## Authors' contributions

AC and RH performed the autopsy. SL followed the patient clinically. The ocular histopathological studies were carried out in FGF's laboratory. AC and FGF conceived of the study, and participated in its design and coordination and helped to draft the manuscript. All authors read and approved the final manuscript.
